# Impacts of COVID-19 Sanitary Cues on Hedonic Appreciation of Foods

**DOI:** 10.3390/foods11121753

**Published:** 2022-06-14

**Authors:** Jarina Gabrielle Aquino Oliveira, Adriana Conceição Soares Sampaio, Olivia Morgan Lapenta

**Affiliations:** Psychological Neuroscience Laboratory, Center for Investigation in Psychology, University of Minho, 4710-057 Braga, Portugal; pg40877@alunos.uminho.pt (J.G.A.O.); adriana.sampaio@psi.uminho.pt (A.C.S.S.)

**Keywords:** COVID-19, multisensory food perception, eating behavior, food choice, dietary preferences

## Abstract

The COVID-19 pandemic led to several lifestyle changes, including eating behavior. Herein, we aimed to evaluate how pandemic-related sanitary cues presented in food videos impact food appraisal and desire to eat, and their priming after-effects on subsequent food pictures presented without such cues. In two online sessions, separated by 4 to 7 days, participants watched either a Non-Pandemic or a Pandemic video of a woman eating, the latter containing sanitary elements adopted during the pandemic. The order of the videos was counterbalanced across participants over the two experimental sessions. Videos were followed by images of food from different categories. After observing both videos and each picture, participants were instructed to evaluate the visual aspect, expected smell and flavor, and rate their desire to eat. Our study demonstrated (1) higher hedonic responses to the Non-Pandemic compared to the Pandemic video, (2) a priming effect showing higher appreciation for sweet foods after the Non-Pandemic compared to the Pandemic video, (3) that food exposure gradually increases one’s desire to eat, but such effects are impacted by pandemic sanitary cues, and (4) greater hedonic responses are given for sweet and high-calorie foods compared to salty and low-calorie ones, irrespective of pandemic priming. Finally, depression and anxiety symptoms were associated with lower smell evaluations only in the Pandemic condition.

## 1. Introduction

More than two years after the SARS-CoV-2 pandemic onset, many countries are still struggling with the long-term consequences caused by the coronavirus disease 19 (COVID-19). To deal with the pandemic and mitigate its spread, a number of social and sanitary measures were imposed, including social distancing, curfews and quarantine, daily use of protective tools (e.g., face masks and face shields), as well as exacerbation of hygienic habits (e.g., constantly washing hands and using hand sanitizer in order to eliminate the virus) [1]. In this sense, the primary impact of COVID-19 was determined by social rules and medical advice, but there are also secondary outcomes in psychological health, as well as in different behaviors, including, but not limited to, eating behavior.

Food perception determines dietary preferences, which impacts people’s physical [2] and mental health [3]. Eating habits are susceptible to change due to sensitivity to environmental cues, among other reasons. In the context of the pandemic, the risk of infection, the dynamics of food distribution and individuals’ psychological state had a major influence on people’s diets worldwide.

During the lockdown established in the first semester of 2020, changes in the quality of food intake, food preferences and purchasing during the social isolation were reported in France [4] and the Netherlands [5]. In Italy, Renzo et al. [6] highlighted the influence of psychological factors on food choice, reporting difficulty in controlling good dietary habits. Additionally, the authors found alterations in eating habits during the pandemic to be positively correlated with different self-reported psychological symptoms.

Furthermore, higher rates of food intake were reported in the United Kingdom [7], whereas a switch onto less healthy behaviors was observed in China, such as the preference for non-fresh foods instead of fresh ones [8]. Moreover, reductions in physical activities and well-being, along with weight gain, were reported in Spain after the outbreak of the COVID-19 pandemic [9].

In turn, Faour-Klingbeil et al. [10] conducted a study in the Arab region, namely in Lebanon, Jordan and Tunisia. Results revealed a high concern of the population of becoming infected with COVID-19 through food. Importantly, these concerns were shown to be influenced not only by the food itself but also by the environment, culture, as well as individual and social beliefs. Therefore, the findings suggest that context and environmental cues surrounding food are linked to the way they are perceived, and as a result, have a major impact on food choices. 

In fact, food perception is known to be multidimensional [11]. Flavor perception and eating experience involve processing and combining information from all of the senses, which are influenced by both intrinsic and extrinsic food features. Further, the sensory inputs are integrated into previous memories (e.g., related to food experiences and/or personal beliefs), building a perception that interferes with individual expectations, and finally shaping food choices and appreciation [12]. For instance, Zellner et al. [13] demonstrated that food rates of pleasantness vary according to the interaction between expectation and real food taste. Moreover, memory processes play an important role in smell/odor perception [14], which can be observed in experiments with taste/odor associative congruence (please see Ref [15] for a review on this topic). 

In sum, while evaluating food, different sources of information are concomitantly accessed and combined to elaborate perception, influencing food choices, preferences and consumption [16]. Given the interference of various factors in the relationship between human beings and food, it is essential to highlight the importance of considering one’s environmental surroundings to better understand how new eating habits arise and how old habits can be modified. 

Considering the context of the COVID-19 pandemic, in particular its impact on mental health and the reorganization of society [17], it is likely that it would influence food perception and eating habits, as reported previously and also demonstrated through online surveys on food purchasing habits [18]. Since food perception is influenced by environmental cues and subjective beliefs [19], and these dimensions are affected by a pandemic [20], it is important to clarify whether hygienic measures implemented to mitigate COVID-19 influenced food appraisal and willingness to eat. This can be achieved by exploring food evaluations during and after the presence vs. the absence of Pandemic sanitary cues.

In order to comprehend how foods presented after Pandemic cues are perceived (i.e., priming effects due to environmental circumstances), one must understand the concept of priming itself. The priming effect is characterized by the modulation of a perceptual process due to a previous exposure of an individual to a stimulus that then affects the assimilation and responses toward a subsequent target stimulus [21].

Once priming alters perception, it is likely to also influence behavior. Regarding studies on eating behavior using priming, Papies and Hamstra [22] conducted an experiment with 156 subjects, for which half of the participants were passively primed with a low-calorie diet poster before being exposed to food that could be eaten ad libitum. Self-regulation in eating volume was shown to be primed by the poster, resulting in lower food consumption when compared to the control condition (no poster), particularly in individuals who self-reported to be restrained eaters. Further, food advertisements have shown to prime food intake of both children and adults [23]. Specifically, snack advertisement priming increased food intake when compared to non-food or healthy food priming [23]. Therefore, it is evident how relevant priming cues affect the behavioral outcomes in food sciences. Furthermore, the duration of the priming effects has been shown proportional to the length of exposure to the priming stimulus [24].

Regarding eating behavior, Papies and Hamstra [21] conducted a behavioral experiment on 156 subjects, for which half of the participants were passively primed with a low-calorie diet poster before being exposed to food that could be eaten ad libitum. Self-regulation in eating volume was shown to be primed by the poster, resulting in lower food consumption when compared to the control condition (no poster), particularly in individuals who self-reported to be restrained eaters. Further, food advertisements have shown to prime food intake of both children and adults [22]. Specifically, snack advertisement priming increased food intake when compared to non-food or healthy food priming [22]. Withal, it is evident how relevant priming cues affect the behavioral outcomes in food sciences.

In sum, food perception is a complex phenomenon influenced by multisensorial inputs, from the food to the environmental cues. Furthermore, emotion states, such as fear [25] and anxiety [26], have been shown to modulate consumer behavior in many ways. Of particular interest, Motoki and colleagues show that negative emotions of high arousal seem to reduce food intake and motivation to eat and that anxiety levels, in particular, increase the appreciation of hedonic vs. healthy foods [27].

Consequently, food behavior was greatly influenced by many contextual changes and individual emotional state resulting from the pandemic and its mitigation. Still, most studies focus on the psychological effects of the pandemic and how eating behaviors were modulated in the context of one’s home. Meanwhile, to the best of our knowledge, such behaviors have not been studied ‘outdoors’, since studies of such nature are less feasible due to lockdown. Herein, we aimed to provide novel insights by evaluating whether pandemic cues, specifically sanitary elements, which consist of resources for protection when individuals are outdoors, would impact hedonic food appreciation. Preliminary findings of our research have shown interesting results and were presented at the 2nd International Electronic Conference on Foods—Future Foods and Food Technologies for a Sustainable World [28]. Here, we extend these findings with a larger sample and further analyses. To achieve the study goals, we presented videos depicting food intake in Pandemic vs. Non-Pandemic situations and collected participants’ responses relating to food appreciation. We were particularly interested in whether each video would lead to differences in the appraisal of the foods presented in the video and further, on the following food pictures presented without pandemic cues. Considering that food appraisal is influenced by context and that COVID-19 is associated with negative emotions and psychological effects, we hypothesized that the presentation of the sanitary cues would be effective in eliciting a pandemic circumstance bias and therefore that the presence of sanitary cues in the Pandemic videos would result in lower ratings for food attractiveness and desire to eat compared to the Non-Pandemic videos (without sanitary cues). These effects were expected both for the video and food pictures evaluations due to video priming. 

## 2. Materials and Methods

### 2.1. General Procedure and Experimental Design

Participants were recruited from University of Minho through the online university system and were compensated with academic credits. Participants were required to be over 18 years of age, have normal or corrected-to-normal vision and no known sensory impairments. 

The present study was conducted in accordance with the Declaration of Helsinki and approved by the University of Minho Ethics Committee for Research in Life and Health Sciences (CEICVS 129/2020). Participants digitally signed an informed consent form before providing any personal data and taking part in the two-day online experiment. Individual anonymity was preserved by generating a personal code using the combination of each individual’s date of birth and their initials. All data for the study were collected using Qualtrics online platform (Qualtrics, Provo, UT, USA), including the questionnaires. Participants were provided with the first and last authors’ emails and with the telephone number of the last author’s office and instructed to contact us in case they faced any issues or had any doubts regarding any of the steps of the procedure.

During the first day, participants completed a demographic questionnaire, along with three self-reported scales: (i) the PHQ-9 for depressive symptomatology [29] adapted for the Portuguese population [30], (ii) the GAD-7 for anxious symptomatology [31] validated in Portuguese [32] and (iii) the LOCES for loss of control over eating [33] validated for the Portuguese population [34]. Participants were also expected to report whether they perceived an increase or decrease in the amount of food intake during the pandemic. Lastly, during both testing days, a COVID-19 questionnaire was completed in order to determine whether participants had a current or past SARS-CoV-2 infection, and if so, whether there were any associated symptoms of taste/smell impairment/loss. 

**Figure 1:**
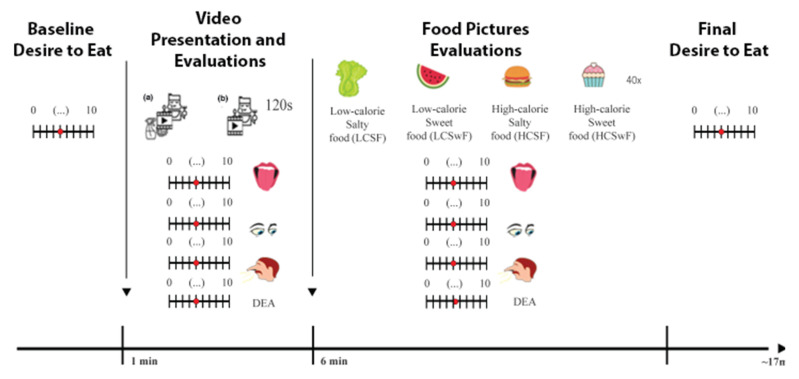
Task design. In the first minute of the task, participants were asked about their desire to eat at that moment. Following the presentation of the video, (**a**) Pandemic and (**b**) Non-Pandemic, participants were asked again to rate their desire to eat, as well as evaluate the visual aspect and expected flavor and smell of the foods presented in the video. Then, participants were presented with several food pictures and were instructed to evaluate them based on visual aspect, expected flavor and smell and their desire to eat each food. After completing the experiment, participants’ desire to eat was reassessed.

Before stimuli exposure, the baseline desire to eat was assessed using an 11-point visual analog scale (VAS) (i.e., 0 = no urge to eat and 10 = extreme urge to eat). On each day, a Pandemic or Non-Pandemic context video was presented, thus generating two experimental conditions and therefore allowing us to have a within-subject design. The order of conditions was randomized and counterbalanced between participants. After the video, participants were again assessed on their desire to eat and were asked to evaluate the foods presented in the video based on the visual aspect and expected smell and flavor using an 11-point VAS (i.e., 0 = extremely unpleasant and 10 = extremely pleasant). After video evaluation, a set of food pictures were presented once in a pseudo-randomized order. The pictures were identical across testing days. Immediately after their presentation, participants were requested to evaluate each food picture using an 11-point VAS, taking into account the visual aspect, expected flavor and smell, and their desire to eat the depicted food. At the end of the task, participants’ ‘desire to eat’ ratings were re-assessed. The experimental design is depicted in Figure 1. Please refer to the Supplementary Material file for specific details on the questions and scales.

### 2.2. Video Stimuli

Videos were recorded in frontal view using an Iphone 7 (Copyright © 2022 Apple Inc. All rights reserved) and were edited using the Adobe Premiere Rush application for iOS (Copyright © 2022 Adobe. All rights reserved). All videos used in this protocol were saved in high-quality, 1080 p resolution and with disabled audio to avoid any unwanted auditory stimuli interference.

The same scenario was depicted in both videos: a white background, a table covered with a checkered white and red tablecloth, a white plate, cutlery and napkins. In each video, a woman consumes food from different categories: Low-Calorie Sweet and Salty, and High-Calorie Sweet and Salty. The video of the Pandemic condition had the following additional elements: a hand sanitizer tube and a light-blue medical face mask. Further, the Pandemic condition video illustrated packaged food, cutlery and napkins (unlike the Non-Pandemic video). Thus, the videos differed only regarding the presence/absence of Pandemic hygienic measures (i.e., sanitary cues, food and cutlery wrapping and individual hygienic procedures (e.g., at the beginning of the video, the woman is wearing a face mask that is only removed after cleaning her hands with alcohol gel prior to food intake)). Both videos are available online elsewhere (https://osf.io/a258r/) (accessed on 26 May 2022).

### 2.3. Food Picture Stimuli

With respect to the visual food stimuli depicted in the food evaluation task, 40 pictures were selected from the Full4Health Image Collection [35], a set of standardized food and non-food images available online at https://osf.io/cx7tp/ (accessed on 2 November 2020). Ten pictures were selected to constitute each of the four categories we created, specifically: for salty low calorie (SaLCF), we selected images 55, 57, 59, 60, 66, 67, 70, 73, 174, 176 (M_kcal/100g_ = 191 SD_kcal/100g_ = 78.13); for salty high calorie (SaHCF), we selected images 12, 130, 131, 190, 198, 317, 318, 321, 322, 370 (M_kcal/100g_ = 285.3 SD_kcal/100g_ = 58.36); for sweet low calorie (SwLCF), we selected images 30, 95, 143, 144, 152, 154, 160, 161, 207, 213 (M_kcal/100g_ = 73.9 SD_kcal/100g_ = 48.05); and for sweet high calorie (SwHCF), we selected images 25, 26, 43, 44, 104, 127, 236, 239, 355, 356 (M_kcal/100g_ = 382.6 SD_kcal/100g_ = 113.41).

### 2.4. Statistical Analysis

Analyses were performed using Jamovi software (The jamovi project, version 1.6.16), considering alpha = 5%. Bonferroni-corrected post hoc analyses were conducted where appropriate. First, we computed the individual mean for each VAS. Importantly, separate analyses were conducted for the picture and video evaluations.

For the pictures, means were computed separately for each food type (i.e., SaLCF, SaHCF, SwLCF, SwHCF). Next, a repeated-measures ANOVA (rmANOVA) was conducted for each perceptual dimension as a dependent variable (i.e., flavor, smell, visual aspect and desire to eat), considering Priming (Pandemic video vs. Non-Pandemic video), Tastant (Sweet vs. Salty) and Calorie (High- vs. Low-calorie) as within-subject factors. 

For the videos, a paired t-test was performed between conditions (Pandemic vs. Non-Pandemic) for each perceptual dimension (i.e., expected flavor, expected smell and visual aspect).

For the general desire to eat, we performed a rmANOVA considering priming (Pandemic vs. Non-Pandemic) and time point (baseline vs. after video vs. after task) as within-subject factors. 

Finally, Pearson correlations were performed between the two psychological scales (i.e., PHQ-9 and GAD-7), as well as between the scales and each of the subjective evaluations (visual aspect, smell, flavor and desire to eat) for each category of the priming videos and food pictures.

## 3. Results

### 3.1. Sample Characterization

Of the 126 individuals that participated in the first day of the study, 41 dropped out prior to completing the second experimental session and were therefore excluded from analysis. The final analyzed sample comprised 85 university students (10 male, 75 female), with a mean age of 20.8 years old (SD = 4.5). Participants’ average weight and height were 60.3 kg (SD = 9.8) and 163 cm (SD = 0.07). Moreover, participants’ average body mass index (BMI) was 22.55 kg/m² (SD = 3.18), with the exception of three obese individuals (BMI > 30). A control analysis excluding participants with obesity showed similar results (please see Supplementary Material for these data). 

Psychological self-reported data pertaining to symptoms of depression and anxiety were sorted by severity (See Table 1). Regarding the depression self-reported ratings (assessed using the PHQ-9), 25 participants did not fit within the diagnosis, while the remaining participants were considered as follows: 3 = severe, 4 = severe/moderate, 18 = moderate and 35 = mild diagnosis. The anxiety ratings (assessed using the GAD-7) were as follows: 14 = severe, 22 = moderate, 28 = mild and 21 = minimal diagnosis.

The LOCES analysis showed that the average total score of our sample was 1.8 (SD = 0.56). Regarding the instrument’s subscales, participants’ mean scores were 1.3 (SD = 0.53) for the cognitive/dissociative factor, 1.9 (SD = 0.86) for the positive/euphoric factor and 2.1 (SD = 0.71) for the behavioral factor. Such numbers show participants’ loss of control over eating to be proportional to the low scores, as 1 is the minimum value of the scale (5 being the highest). Thus, the results indicate low potential for loss of control over eating.

Regarding COVID-19 infection, one participant reported being infected by COVID-19 at the time of testing but without a loss of taste and smell. Another 12 participants reported being infected in the past, of which 8 communicated having associated (but fully recovered) taste and smell symptoms. Finally, 51 participants reported no changes in food consumption during the pandemic, whereas 23 reported increased food consumption and 11 reported decreased food consumption compared to the level of food consumption prior to the pandemic.

### 3.2. Food Pictures Evaluation

#### 3.2.1. Visual Aspect

**Figure 2:**
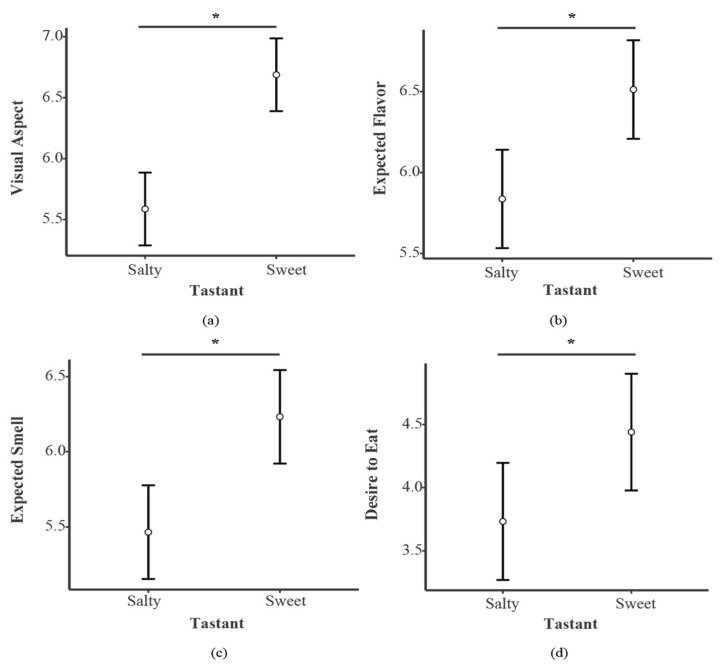
Main results for Tastant in each evaluated dimension, i.e., (**a**) Visual Aspect, (**b**) Expected Flavor, (**c**) Expected Smell and (**d**) Desire to Eat. Bars indicate confidence intervals (95%). * *p* < 0.05.

**Figure 3:**
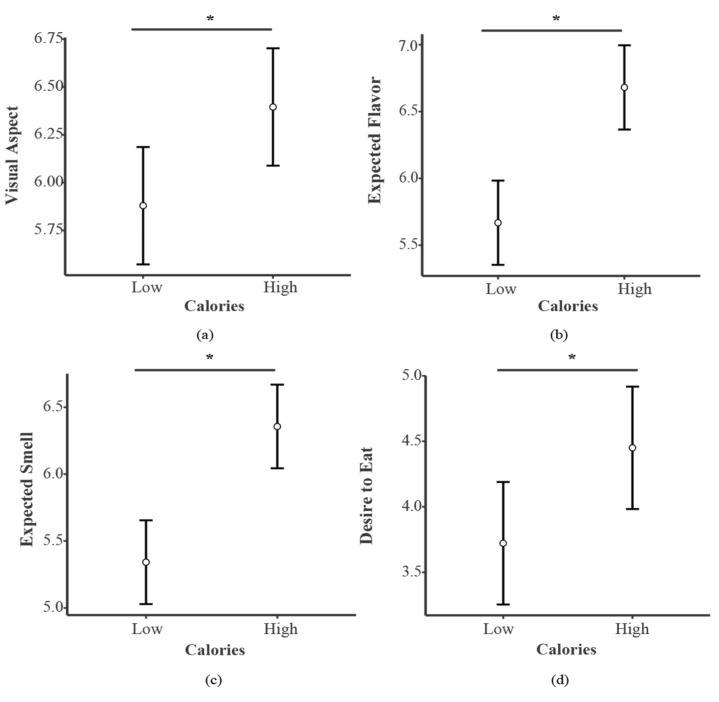
Main results for Calories in each evaluated dimension, i.e., (**a**) Visual Aspect, (**b**) Expected Flavor, (**c**) Expected Smell and (**d**) Desire to Eat. Bars indicate confidence intervals (95%). * *p* < 0.05.

The rmANOVA for the visual aspect evaluations showed a main effect for Tastant (F_1,84_ = 83.521, *p* < 0.001, η^2^_p_ = 0.499) (see Figure 2, panel a) and Calories (F_1,84_ = 13.39, *p* < 0.001, η^2^_p_ = 0.137) (see Figure 3, panel a). Specifically, Sweet foods (SwF) received higher scores compared to Salty foods (SaF), and High-calorie foods (HCF) received higher scores compared to Low-calorie foods (LCF). No main effect of priming was found (F_1,84_ = 0.541, *p* = 0.464, η^2^_p_ = 0.006). 

Further, we found interaction effects for Priming*Calories (F_1,84_ = 10.826, *p* < 0.001, η^2^_p_ = 0.114) and Priming*Calories*Tastant (F_1,84_ = 7.019, *p* < 0.010, η^2^_p_ = 0.077). Bonferroni post hoc analysis on the Priming*Calories effect revealed that LCF received lower ratings compared to HCF in both Pandemic (*p* = 0.072) and Non-Pandemic conditions (*p* < 0.001). In turn, post hoc analysis on the Priming*Calories*Tastant interaction showed the same effects for Tastant and Calories, across both Pandemic and Non-Pandemic conditions.

#### 3.2.2. Smell

With respect to the expected smell evaluations, the rmANOVA revealed main effects for Tastant (F_1,84_ = 30.534, *p* < 0.001, η^2^_p_ = 0.267) (see Figure 2, panel c) and Calories (F_1,84_ = 50.647, *p* < 0.001, η^2^_p_ = 0.376) (see Figure 3, panel c). SwF and HCF obtained higher rates compared to SaF and LCF, respectively. No main effect was found for Priming (F_1,84_ = 0.116, *p* = 0.733, η^2^_p_ = 0.001). 

Interaction effects were found for Tastant*Calories (F_1,84_ = 24.410, *p* < 0.001, η^2^_p_ = 0.225) and Tastant*Calories*Priming (F_1,84_ = 6.417, *p* = 0.013, η^2^_p_ = 0.071). Bonferroni post hoc analysis on the Tastant*Calories interaction showed that SwHCF and SaHCF received higher ratings when compared to SwLCF (*p* = 0.012) SaLCF, respectively (*p* < 0.001). 

Bonferroni post hoc analysis on the triple interaction showed that SaLCF presented in the Pandemic condition received lower ratings when compared to SaHCF (*p* < 0.001) and SwLCF (*p* < 0.001). SwLCF had lower ratings when compared to SwHCF in the Pandemic condition (*p* = 0.022). When examining the triple interaction, the same main effects for Tastant and Calories were present within the Pandemic and Non-Pandemic conditions; however, no other meaningful comparisons were significant. 

#### 3.2.3. Flavor

For flavor expectation, the rmANOVA showed main effects for Tastant (F_1,84_ = 21.438, *p* < 0.001, η^2^_p_ = 0.203) (see Figure 2, panel b) and Calories (F_1,84_ = 35.762, *p* < 0.001, η^2^_p_ = 0.299) (see Figure 3, panel b). SwF and HCF received higher scores when compared to SaF and LCF. No main effect was found for Priming (F_1,84_ = 3.614, *p* = 0.061, η^2^_p_ = 0.041).

**Figure 4:**
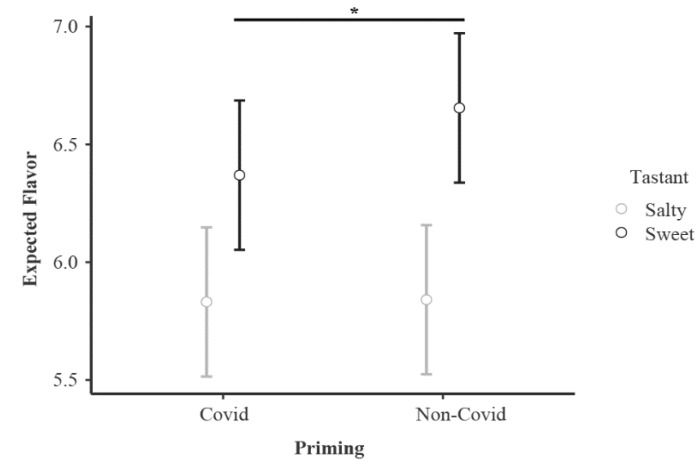
Priming effect observed for sweet food (vs. salty) in flavor expectation. Interaction effect between Priming video and Tastant is depicted. Graph depicts the mean value for flavor expectation. Bars indicate confidence intervals (95%). * *p* < 0.05.

There were also interaction effects for Priming*Tastant (F_1,84_ = 7.969, *p* = 0.006, η^2^_p_ = 0.087), Priming*Calories (F_1,84_ = 7.668, *p* = 0.007, η^2^_p_ = 0.084) and Tastant*Calories (F_1,84_ = 18.601, *p* < 0.001, η^2^_p_ = 0.181). Bonferroni post hoc analysis on the Priming*Tastant interaction revealed that Non-Pandemic SaF received lower ratings when compared to SwF in the same condition (*p* < 0.001). Furthermore, SwF received higher ratings compared to SaF in the Pandemic condition (*p* = 0.004). SwF in the Non-Pandemic condition received higher rating compared to SwF in the Pandemic condition (*p* = 0.013) (Figure 4).

Regarding the Priming*Calories interaction, Bonferroni post hoc analysis showed that HCF were scored higher when compared to LCF in both the Pandemic (*p* < 0.001) and the Non-Pandemic conditions (*p* < 0.001). Regarding the Tastant*Calories interaction, SaLCF scored less compared to SwLCF (*p* < 0.001) and SaHCF (*p* < 0.001).

#### 3.2.4. Desire to Eat Evaluation

The rmANOVA evaluation revealed main effects for Tastant (F_1,84_ = 18.317, *p* < 0.001, η^2^_p_ = 0.179) (Figure 2, panel d) and Calories (F_1,84_ = 16.729, *p* < 0.001, η^2^_p_ = 0.166) (Figure 3, panel d). HCF and SwF presented higher scores compared to LCF and SaF, respectively. No main effect was found for Priming (F_1,84_ = 1.109, *p* = 0.295, η^2^_p_ = 0.013).

Interaction effects were found for Tastant*Priming (F_1,84_ = 7.381, *p* = 0.008, η^2^_p_ = 0.081) and Tastant*Calories (F_1,84_ = 18.086, *p* < 0.001, η^2^_p_ = 0.177). Bonferroni post hoc analysis for the Tastant*Priming interaction demonstrated that for both Pandemic and Non-Pandemic conditions, SwF received higher scores compared to SaF (*p* = 0.038; *p* < 0.001, respectively). Regarding the Tastant*Calories interaction, SaLCF scored less when compared to SaHCF (*p* < 0.001) and SwLCF (*p* < 0.001).

Finally, a Pearson correlation between the PHQ-9 and the food picture evaluations showed a significant negative correlation between depression symptomatology and visual aspect (r = −0.24, *p* = 0.025), flavor (r = −0.25, *p* = 0.023) and smell (r = −0.25, *p* = 0.021) ratings of SaLC foods in the Pandemic condition. No significant correlation was found between the PHQ-9 and GAD-7 and participants’ desire to eat.

### 3.3. Video Evaluation

**Figure 5:**
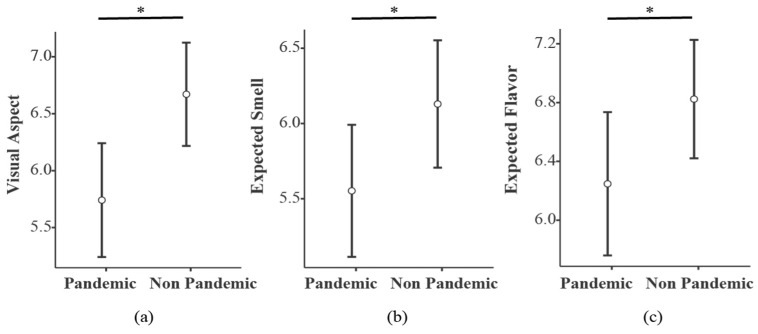
Comparison between video condition evaluations of expected flavor, smell and visual aspect. (**a**) Flavor expectation compared between Pandemic (M = 6.25, SD = 2.29) and Non-Pandemic (M = 6.82, SD = 1.90) conditions showed higher scores for the Non-Pandemic condition; (**b**) Visual aspect between conditions showed higher rates for the Non-Pandemic condition (M = 6.67, SD = 2.13) compared to the Pandemic condition (M = 5.77, SD = 2.35); and (**c**) Smell expectation, comparing the Pandemic condition (M = 5.55, SD = 2.06) to the Non-Pandemic condition (M = 6.13, SD = 1.99), showed higher scores for the Non-Pandemic condition. The letter ‘C’ on the plots stands for Pandemic condition, while the letters ‘NC’ stand for Non-Pandemic condition. Confidence interval bars are also depicted (95%). (*) *p* = <0.05.

T-tests (Figure 5) showed higher scores for the Non-Pandemic compared to the Pandemic condition for visual aspect (t = −3.56, *p* < 0.001, d = −0.386), smell (t = −2.26, *p* = 0.026, d = −246) and flavor expectation (t = −2.50, *p* = 0.015, d = −271). 

### 3.4. Desire to Eat Evaluation

**Figure 6:**
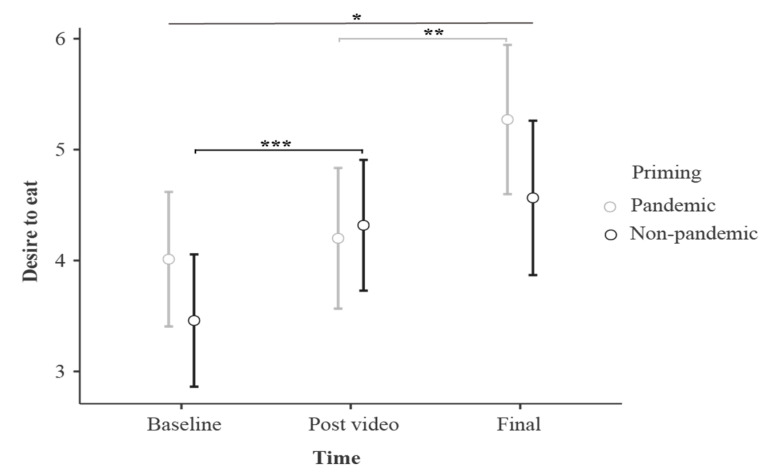
Desire to eat at the different time points of the experiment. The image depicts the main effects of desire to eat throughout the task, considering the different time points. Namely, the (*) Final assessment revealed higher desire to eat ratings when compared to the Baseline in both Pandemic and Non-Pandemic conditions, (**) the Final evaluation showed higher desire to eat ratings when compared to the Post-video evaluation in the Pandemic condition, and (***) the Post-video revealed higher desire to eat ratings when compared to baseline assessment in the Non-Pandemic condition. Bars indicate confidence intervals (95%), *p* < 0.001.

The rmANOVA for the desire to eat evaluations showed a main effect of Time (F_2_._168_ = 17.11, *p* < 0.001, η^2^_p_ = 0.169), revealing an increase for the desire to eat as the experiment progressed. Specifically, the final desire to eat rating was higher when compared to the Baseline assessment (*p* < 0.001) and Post-priming evaluation (*p* = 0.004). Further, Post-priming evaluations received higher ratings compared to Baseline evaluations (*p* = 0.032) (Figure 6). 

Furthermore, an interaction effect was found for Time*Priming (F_2_._168_ = 5.17, *p* = 0.007, η^2^_p_ = 0.058). Bonferroni post hoc analysis showed that the Final desire to eat rating in the Pandemic condition was higher when compared to the Baseline (*p* < 0.001) and also the Post-priming desire to eat rating (*p* < 0.001), whereas in the Non-Pandemic condition, the Final evaluation was only greater when compared to the Baseline (*p* < 0.001). No main effect of Priming (F_1.84_ = 1.28, *p* = 0.262, η^2^_p_ = 0.015) was found.

### 3.5. Correlational Analyses 

Pearson correlations between the PHQ-9 and GAD-7 scales and the video subjective evaluations revealed a negative correlation between the smell evaluation and the PHQ-9 (*r* = −0.25, *p* = 0.019) and GAD-7 (*r* = −0.28, *p* = 0.010) in the Pandemic condition. No other significant correlations were found.

## 4. Discussion

We proposed an innovative and authorial online task to explore how pandemic-related sanitary cues impact the hedonic perception (i.e., visual attractiveness, expectation of flavor/smell and desire to eat) of sweet and salty foods with low and high energetic content. The design was built specifically for investigating how the pandemic context (cued using COVID-19 sanitary cues) affects food appraisal and eating behavior. Specifically, we compared participants’ desire to eat and subjective hedonic evaluations of foods presented in videos depicting Pandemic and Non-Pandemic scenarios. We then explored possible priming effects by asking participants to evaluate food pictures (without sanitary cues) presented after the videos.

Within our main outcomes, we observed that flavor and smell expectation and visual aspect of the foods in the Non-Pandemic condition received higher scores compared to the Pandemic condition. Furthermore, in line with the notion that exposure to food or food cues induces craving (i.e., urge to eat in humans [36]), the desire to eat over time ratings showed that the final urge to eat was higher when compared to the baseline in both experimental conditions. 

Interestingly, when comparing the desire to eat ratings after the video with the initial assessment, we observed that only the Non-Pandemic video led to increased ratings compared to the baseline. Such effects likely arise from the aversive/negative associations with the Pandemic condition, which was also associated with lower hedonic appreciation of the foods. Therefore, the negative aspects of the video counterbalance the expected food cue effect of increasing the desire to eat. Importantly, while both videos were almost identical in content (i.e., foods depicted), they differed only in terms of the Pandemic and Non-Pandemic sanitary cues, as well as the hygienic measures taken before eating the foods. Thus, the video evaluation findings are aligned with our initial hypothesis. 

The fact that participants’ desire to eat at the end of the task was higher in both conditions when compared to the baseline (thus diluting the negative effects of the Pandemic cues) is in accordance with Dijksterhuis and Van Knippenberg [24]. In their study, they showed that priming effects vary with duration of exposure and time after exposure. Specifically, priming effects are stronger soon after the priming stimulus. Thus, in the present study, the increased craving at the end of both conditions seems to be due to the repetitive presentation of the food images. 

Still, the craving and hedonic ratings of the foods following the presentation of the video, as well as the priming effect, on the evaluation of the sweet food images (i.e., higher flavor expectations in the Pandemic compared to the Non-Pandemic condition) are in line with previous studies, showing that participants are more likely to positively evaluate foods in the absence of negative events [37]. 

Considering the negative impact that the COVID-19 pandemic had on psychological, social and health aspects of life [17], we expected food hedonic appreciation and desire to eat to be lower in the Pandemic video compared to the Non-pandemic video, for both the actual video and the food images presented after the video. In fact, sweet foods in Non-Pandemic condition received higher scores for flavor expectation when compared to Pandemic condition. The sanitary cues primed along food exposure have previously been shown to diminish unhealthy choices [38]. Thus, our study results are partly in accordance with the initial hypothesis. Interestingly, our priming effect observed in the food picture evaluations occurred only for the most salient category (i.e., sweet foods). Specifically, sweet foods received higher ratings in the Non-Pandemic condition compared to the pandemic condition. Not only did the video illustrate the sanitary measures associated with food consumption, but it also provided an accurate representation of the pandemic scenario, touching on the negative burden that the pandemic has on mental health. Different levels of psychological distress are known to modify individuals’ relationship with food [39,40,41]). The effects of non-conscious odor, visual and auditory priming are observed in behavioral and lexical tasks investigating food perception [22,42,43], which supports our finding that sweet foods received higher evaluations when participants were primed with Non-Pandemic-related situations. 

Our study is limited by not verifying participants’ explicit awareness of the priming. We used an implicit priming approach (i.e., we did not instruct participants regarding the different conditions nor ask if they presumed that each video was supposed to affect their evaluation of the following food image evaluations). Such information could be insightful for future studies to control personal biases and to investigate how awareness potentially interferes with these evaluations. Moreover, as our main goal was to compare how a pandemic-related vs. a non-pandemic scenario would affect food perception and appreciation, we used COVID-related sanitary cues to incite the COVID-related scenario; however, the absence of a third condition using sanitary cues unrelated to COVID-19 limits our interpretations regarding the disentangling of potential effects from the general sanitary elements on food appreciation.

In addition to the main findings regarding condition, we also observed the effects of food type. In line with our prediction of differential evaluations regarding the caloric content and tastant, we demonstrated the salience of sweet over salty foods and of high-calorie over low-calorie foods in all dimensions (i.e., smell and flavor expectation, visual aspect and desire to eat). Sweet (vs. salty) and high-calorie (vs. low-calorie) foods usually elicit greater hedonic responses. Sweet preference or attraction is present even in the early stages of human life [44,45,46]. Moreover, high-calorie foods (usually highly processed and rich in fat) have been reported to trigger brain areas connected to the reward system [47], which are also linked to addictive behaviors [48]. Further, preferences for high-density caloric foods are aligned with the increased craving and consumption of sweet and savory snacks associated with the COVID-19 pandemic [7], as well as the higher volume of calorie consumption that can also be related to the maladaptive coping strategies during the pandemic [49,50]. 

The visual information of food plays an important role in its perception and appreciation [46,51], including in digital experiences [52], which has been shown at behavioral and physiological (e.g., heart rate and skin conductance) levels [53]. Thus, conducting an online experiment (which has been used in previous studies [54,55]) granted us the opportunity to explore the link between food perception and pandemic sanitary cues in the context of the pandemic. However, such an approach does not allow us to investigate the robustness of the food category and priming effects toward real food, which would represent a more ecological setting, and also the actual evaluation of smell and flavor beyond its expectation. Future studies, carried out in direct contact with participants in a laboratory or controlled environment, would fill such gaps associated with online data collection.

Finally, regarding the correlations between the depression and anxiety measures and the subjective evaluations, we found a negative correlation between the smell expectation and the depressive symptomatology only for the video evaluation in the Pandemic condition. In line with this finding, Choi et al. [56] showed high prevalence of participants reporting symptoms of anxiety and depression in studies related to COVID-19. In turn, although previous findings report higher consumption and greater food appeal for individuals with negative psychological burden [57,58], our study suggests lower hedonic appreciation of food in the Pandemic scenario. 

It is important to acknowledge that our study is limited to an online format, while previous studies presenting participants with real food [59,60] allow measuring actual smell and flavor appreciation. As a result, we cannot assure that such effects would be the same in the presence of actual food. Still, it is remarkable that the hygienic cues illustrated in our videos (i.e., hand sanitizing and the use of a face mask) evoked negative triggers to one’s mental health, ultimately affecting food perception and potentially food behavior [17].

Herein, we presented new insights into the impacts of COVID-19 on food behavior, which has significance due to the scale of its impact worldwide. In summary, we identified that the presence of COVID-19 hygienic cues led to reduced cravings, as well as lower hedonic appreciation of the visual aspect, food smell and flavor expectations. Furthermore, sweet food pictures presented after sanitary pandemic cues were rated significantly lower in comparison to the Non-Pandemic condition. Additionally, irrespective of the video condition, we observed higher ratings for sweets and high-calorie foods compared to salty and low-calorie ones, which was in line with our hypothesis and previous literature. Future studies in this field could benefit from coupling physiological assessments and behavioral/psychological data. Neurophysiological information (e.g., brain activity, skin conductance and eye-tracking recordings) could provide additional information relating to different dimensions, affording a better understanding of which brain areas are involved with the observed effects (e.g., the levels of arousal evoked by different categories of stimuli, whether priming changes the way food is tracked and whether different brain areas are activated according to food type and priming condition).

## 5. Conclusions

In conclusion, our data support previous findings that (i) sweet and high-calorie foods have greater attractiveness and better ratings when compared to salty and low-calorie foods, and (ii) food exposure increases the desire to eat. In addition, the study provided new insights, demonstrating that hygienic measures of COVID-19 may elicit lower smell assessment and desire to eat, as shown by our findings in the Non-Pandemic and Pandemic video evaluations. Furthermore, a priming effect was observed for sweet food picture evaluations but not for the other food categories. This finding points to changes in food behavior, although this might be observable only in more salient food categories. 

When combined with previous findings, our results point to a possible ambivalence toward the learned associations related to the COVID-19 pandemic. This statement stems from our findings that exposure to hygiene measures adopted in the pandemic may affect food perception by reducing the hedonic appreciation of foods, and thus, consumption, whereas previous studies have shown psychological effects of the pandemic to result in increased food intake. Additionally, our findings suggest that the symptoms of depression and anxiety are associated with negative effects of the hygiene measures adopted in the COVID-19 pandemic. Thus, further studies are needed to better understand how individuals’ psychological states modulate food choices and consumption with respect to different scenarios and experimental settings. Finally, because the videos suggest a priming effect only for one food category, further studies should continue exploring this topic in order to clarify whether this finding is due to the salience of sweet food stimuli or to a low sensitivity of our task and/or the priming video. 

## Figures and Tables

**Table 1:** Psychological data description.

PHQ-9	**Did Not Fit**	**Severe**	**Severe/Moderate**	**Moderate**	**Mild**
	25	3	4	18	35
GAD-7	**-**	**Severe**	**Moderate**	**Minimal**	**Mild**
		14	22	28	21

## Data Availability

All data are held in a public repository, available at OSF database (URL access: https://osf.io/a258r/) (accessed on 26 May 2022).

## Supplementary Materials

The following supporting information can be downloaded at: https://www.mdpi.com/article/10.3390/foods11121753/s1, Task subjective evaluation scales; Table S1: Food Picture Evaluations—Main Effects Table S2: Food Picture Evaluations—Interaction Effects; Table S3: T-test Results for video evaluations (excluding obese participants); Table S4: Desire to Eat in Different Time points -Main Effects (excluding obese participants); Table S5: Desire to Eat in Different Time points—Post hoc on interaction effects (excluding obese participants); Figure S1: Desire to Eat in the Different Time Points of Evaluation (excluding obese participants).
