# Supplementary material for: Impacts of COVID-19 Sanitary Cues on Hedonic Appreciation of Foods
Source: Foods. 2022 Jun 14;11(12):1753. doi: 10.3390/foods11121753 (PMC9222980; doi:10.3390/foods11121753)
Supplement: Supplementary file 1 [file foods-11-01753-s001.zip › foods-1695414-supplementary.pdf]

Supplementary Material

1. Task Scales Description

1.1 Baseline Desire to Eat Evaluation

Use the scale below and answer according to the answer that best describes your desire to eat at the moment.

**0**	**1**	**2**	**3**	**4**	**5**	**6**	**7**	**8**	**9**	**10**

No Urge to Eat **Extreme Urge to Eat**

1.2 Video Evaluation

Consider the foods and situations presented in the video to answer the scales regarding how much you desire to eat the presented foods at the moment and how pleasant was the visual aspect, smell and flavor of the foods. Some food characteristics such as smell and flavor cannot be perceived by video, so we ask you to respond based on your expectation about these attributes.

Visual Aspect/Attractiveness

**0**	**1**	**2**	**3**	**4**	**5**	**6**	**7**	**8**	**9**	**10**

Extremely Unpleasant **Extremely Pleasant**

Smell Expectation

**0**	**1**	**2**	**3**	**4**	**5**	**6**	**7**	**8**	**9**	**10**

Extremely Unpleasant **Extremely Pleasant**

Flavor Expectation

0 1 2 3 4 5 6 7 8 9 10

Extremely Unpleasant **Extremely Pleasant**

Use the scale below and answer according to the answer that best describes your desire to eat the presented foods at the moment.

0 1 2 3 4 5 6 7 8 9 10

No Urge to Eat **Extreme Urge to Eat**

1.3 Food Pictures Evaluations

Consider the food picture immediately presented before each scale set to answer the scales. Namely, how much you desire to eat the food at the moment; how pleasant is the visual aspect of the food; and regarding smell and flavor, that cannot be perceived by the picture, we ask you to respond based on your expectation of pleasantness regarding such attributes.

Use the scales below to answer according to what best describes:

Your desire to eat this food at the moment.

0 1 2 3 4 5 6 7 8 9 10

No Urge to Eat **Extreme Urge to Eat**

Visual Aspect/Attractiveness

0 1 2 3 4 5 6 7 8 9 10

Extremely Unpleasant **Extremely Pleasant**

Smell Expectation

0 1 2 3 4 5 6 7 8 9 10

Extremely Unpleasant **Extremely Pleasant**

Flavor Expectation

0 1 2 3 4 5 6 7 8 9 10

Extremely Unpleasant **Extremely Pleasant**

1.4 Final Desire to Eat Evaluation

Use the scale below and answer according to the answer that best describes your desire to eat at the moment

0 1 2 3 4 5 6 7 8 9 10

No Urge to Eat **Extreme Urge to Eat**

2. Table S1 depicts main results for food pictures regarding all subjective evaluations, i.e. Visual Aspect, Expected Smell, Expected Flavor and Desire to Eat, excluding participants with obesity. As can be seen in Table S2 the observed significant results are in the same direction as in the analysis performed with the obese participants.

Table S1. Food Picture Evaluations - Main Effects.

Dimensions		$F_{(1,81)}$	p	η^2p
Visual Aspect	Priming	0.87	0.354	0.01
	Tastant	78.09	<0.001	0.49
	Calories	13.41	<0.001	0.14
	Priming	0.61	0.438	0.01

Expected Smell	Tastant	27.77	<0.001	0.26
	Calories	50.5	<0.001	0.38
Expected Flavor	Priming	3.99	0.049	0.05
	Tastant	18.99	<0.001	0.19
	Calories	36.08	<0.001	0.31
Desire to Eat	Priming	0.83	0.364	0.01
	Tastant	15.45	<0.001	0.16
	Calories	17.48	<0.001	0.018

Table S2. Food Pictures Evaluations - Interaction Effects.				
Dimensions			$t_{(1,81)}$	$p_{bonferroni}$
Visual Aspect	Tastant*Calories	Sweet high > Salty high	-7.63	<0.001
		Sweet low > Salty low	-6.94	<0.001
	Tastant*Priming	Sweet Pandemic > Salty Pandemic	-8.05	<0.001
		Sweet N-Pandemic > Salty N-Pandemic	-8.88	<0.001
	Priming*Calories	High N-Pandemic > Low N-Pandemic	-4.29	<0.001
		High Pandemic > Low Pandemic	-2.7	0.05

	Tastant*Calories* Priming	Sweet high Pandemic. ><br>Salty high Pandemic	-7.03	<0.001
		Sweet low Pandemic ><br>Salty low Pandemic	-5.73	<0.001
		Sweet high N-Pandemic ><br>Salty high N-Pandemic	-7.29	<0.001
		Sweet low N-Pandemic ><br>Salty low N-Pandemic	-7.33	<0.001
Expected Smell	Tastant*Calories	Sweet high ><br>Salty high	-3.61	0.003
		Sweet low ><br>Salty low	-6.47	<0.001
		Salty high ><br>Salty Low	-7.85	<0.001
	Tastant*Priming	Sweet Non Pandemic ><br>Sweet Pandemic	-5.01	<0.001
		Sweet N-Pandemic ><br>Salty N-Pandemic	-4.98	<0.001
	Priming*Calories	High N-Pandemic ><br>Low N-Pandemic	-6.84	<0.001
		High Pandemic ><br>Low Pandemic	-.6.87	<0.001
	Tastant*Calories* Priming	Salty high Pandemic<br>Salty low Pandemic	-6.93	<0.001
		Sweet low Pandemic ><br>Salty low Pandemic	-5.61	<0.001

		Sweet high Pandemic > Sweet low Pandemic	-3.8	0.008
		Salty high N. Pandemic > Salty low N. Pandemic	-8.15	<0.001
		Sweet low N. Pandemic > Salty low N. Pandemic	-6.54	<0.001
Expected Flavor	Tastant*Calories	Salty high > Salty low	-7.96	<0.001
		Sweet low > Salty low	-5.37	<0.001
	Tastant*Priming	Sweet Pandemic > Salty Pandemic	-3.35	0.007
		Sweet N-Pandemic > Salty N-Pandemic	-4.98	<0.001
	Priming*Calories	High N-Pandemic > Low N-Pandemic	-6.62	<0.001
		High Pandemic > Low Pandemic	-5.16	<0.001
		High N-Pandemic > High Pandemic	-3.01	0.021
	Tastant*Calories* Priming	Salty high Pandemic Salty low Pandemic	-6.7	<0.001
		Sweet low Pandemic > Salty low Pandemic	-5.12	<0.001
		Sweet high N-Pandemic > Sweet high Pandemic	-3.95	0.003

		Sweet low N. Pandemic > Salty low N. Pandemic	-6.12	<0.001
		Salty high N. Pandemic > Salty low N. Pandemic	-7.27	<0.001
Desire to Eat	Tastant*Calories	Salty high > Salty low	-6.05	<0.001
		Sweet low > Salty low	-4.73	<0.001
	Tastant*Priming	Sweet N-Pandemic > Salty N-Pandemic	-4.87	<0.001
	Priming*Calories	High N-Pandemic > Low N-Pandemic	-4.37	<0.001
		High Pandemic > Low Pandemic	-3.52	0.004
	Tastant*Calories* Priming	Salty high Pandemic Salty low Pandemic	-4.89	<0.001
		Sweet low Pandemic > Salty low Pandemic	-3.82	0.005
		Salty high N. Pandemic > Salty low N. Pandemic	-5.61	<0.001
		Sweet low N. Pandemic > Salty low N. Pandemic	-6.15	<0.001

3. Pairwise comparison for all dimensions evaluated post video comparing Pandemic and Non-pandemic conditions excluding Participants with Obesity (n = 82).

Table S3. T-test Results for video evaluations (excluding obese participants).				
Comparisons		$t_{(1,81)}$	p	Cohen's d
Pandemic Visual Aspect	Non-Pandemic Visual Aspect	3.53	<0.001	0.39
Pandemic Expected Smell	Non-Pandemic Expected Smell	2.27	0.026	0.25
Pandemic Expected Flavor	Non-Pandemic Expected Flavor	2.3	0.015	0.27

4. Analysis on Desire to Eat at different time-points of the task, excluding obese participants, showed similar results as when including these participats in the analyses. These results are described in Tables S4 and S5 and also depicted at Figure S1.

Table S4. Desire to Eat in Different Time-points -Main Effects (excluding obese participants).				
			p	η^2p
Desire to Eat	Time	$F_{(1,81)}$= 15.72	<0.001	0.16
	Priming	$F_{(1,81)}$= 1.01	0.318	0.01
	Time*Priming	$F_{(2,162)}$ = 4.82	0.009	0.06

Table S5. Desire to Eat in Different Time-points - Post hoc on interaction effects (excluding

obese participants).				
Dimensions			$t_{(2,1.92)}$	$p_{bonferroni}$
Desire to Eat	Time*Priming	Final DtE Pandemic> Inicial DtE Pandemic	-4.93	<0.001
		Final DtE N-Pandemic> Post video DtE N-Pandemic	-3.56	0.006
		Inicial DtE N-Pandemic > Final DtE N-Pandemic	-4.39	<0.001
		Final DtE Pandemic> Post video DtE Pandemic	-4.05	<0.001

Figure S1. *Desire to Eat in the Different Time-Points of Evaluation (excluding obese participants).*

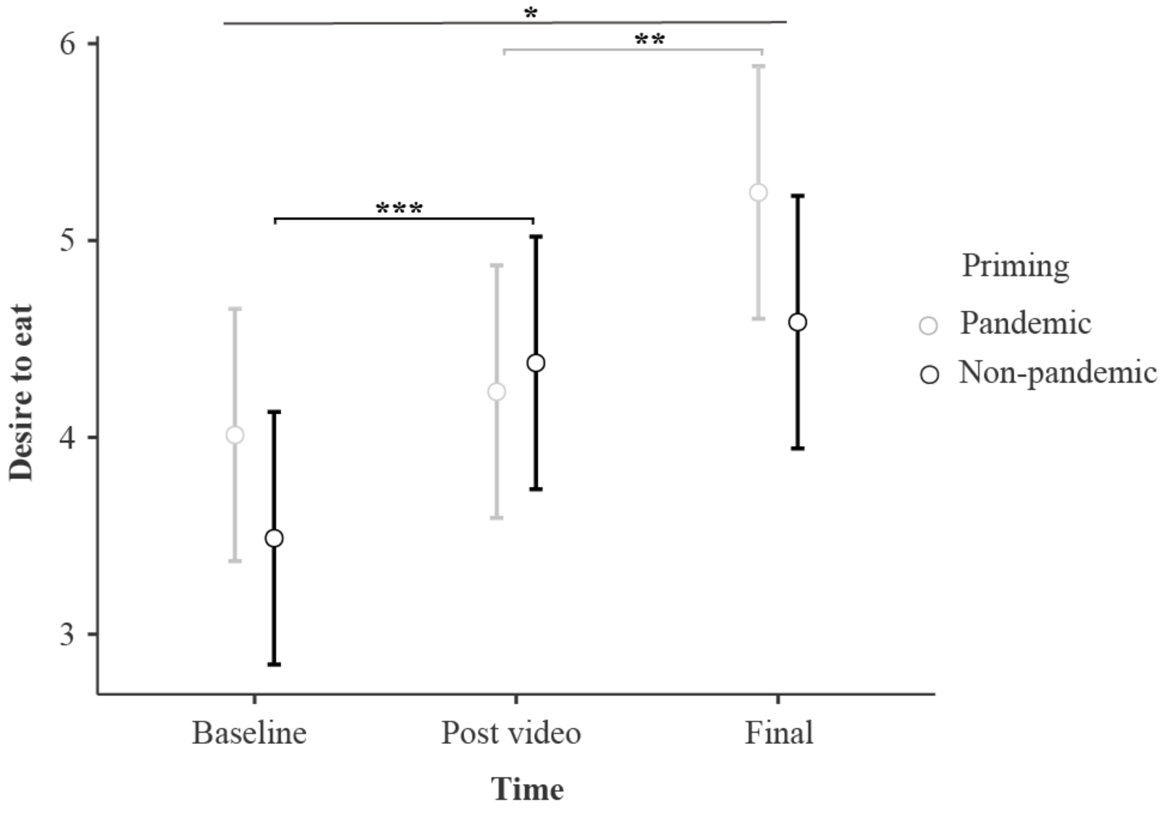

Note. Image depicting the main effects of desire to eat along the task considering different time-points. Namely, the (*) Final assessment, after the end of the task showed higher desire to eat when compared to the assessment before the procedures on both Pandemic condition and Non-pandemic conditions, (**) postpriming evaluation at Pandemic condition showed higher desire to eat when compared to the after video evaluation, (***) and post priming at Non-Pandemic condition compared to baseline assessment on the same condition. Bars indicate confidence intervals (95%) $p < 0.05$.
